# Nanosecond-pulsed DBD plasma treatment on human leukaemia Jurkat cells and monoblastic U937 cells in vitro

**DOI:** 10.1038/s41598-022-10056-8

**Published:** 2022-04-15

**Authors:** Rasool Erfani, Cameron Carmichael, Thea Sofokleous, Qiuyu Wang

**Affiliations:** 1 Department of Engineering, Manchester Metropolitan University, Manchester, M1 5GD UK; 2 Department of Civil, Environmental and Geomatic Engineering, UCL, London, WC1E 6BT UK; 3 Department of Life Sciences, Manchester Metropolitan University, Manchester, M1 5GD UK

**Keywords:** Cancer, Engineering, Physics, Plasma physics

## Abstract

Plasma therapy offers an exciting and novel way of cancer treatment. Specifically, it is shown that Jurkat death rates are closely governed by the plasma treatment time. However, apart from time, alterations to different parameters of treatment process may yield better results. Here, Dielectric barrier discharge (DBD) reactors excited by a nanosecond-pulse energy source are used to investigate cell viability for longer exposure times as well as the effects of polarity of reactor on treatment. Plasma discharge regimes are discussed and assessed using imaging and thermal imaging methods. We found that by changing the polarity of reactor i.e. changing the direction of plasma discharge, the plasma discharge regime changes influencing directly the effectiveness of treatment. Our results showed that ns-DBD− reactor could induce both apoptosis and necrosis of human Jurkat and U937 cells, and this cytotoxic effect of plasma was not completely antagonized by N-acetyl cysteine. It indicates that plasma could induce ROS-independent cell death. Gene expression analyses revealed that p53, BAD, BID and caspase 9 may play vital roles in plasma caused cell death. In addition, our findings demonstrate how different parameters can influence the effectiveness of our reactors. Our assay reveals the custom ability nature of plasma reactors for hematologic cancer therapy and our findings can be used for further development of such reactors using multi-objective optimisation techniques.

## Introduction

Dielectric barrier discharge (DBD) reactor generates non-thermal plasma via the ionisation of the surrounding gas by means of the electrodes and dielectric material^1–3^. Plasma generation in gas, liquid, or at the gas-liquid interface initiates chemical and physical processes. Chemically, it leads to the formation of active oxidizing species, such as radicals (e.g. H$^{\bullet }$, O$^{\bullet }$, OH$^{\bullet }$) and molecules (e.g. H$_2$O$_2$, O$_3$, N$_2$O, NO$^{-}_{2}$, NO$^{-}_{3}$). Physically, it causes shock waves and intense UV light emission^4–8^. In treatment in vitro, intracellular DBD-originated reactive species proved to have main contributing factors in DNA damage^9,10^. These series of actions break the cell membrane which showed their promising apoptotic effects on cancer cell lines of lung^11^, brain^12^, gynecological^13^, breast^14^, colorectal^15,16^, blood^17^ and skin^18^. Turrini et al.^19^ proved that the DBD plasma reactors effectively induces apoptosis in Jurkat cells due to increased generation of reactive oxygen and nitrogen species (RONS) levels. It is believed that non-thermal plasma promotes oxidative stress, resulting in increased intracellular RONS levels, including, hydroxyl radicals ($^{\bullet }$OH), atomic nitrogen (N) and oxygen (O), nitric oxide (NO) and superoxide (O$_2^-$)^20^, and subsequently cell death. However, those levels are restored to normal pre-treatment levels days after treatment^21^. In addition, research is signifying that non-thermal plasma induces the generation of hydrogen peroxide (H$_2$O$_2$) which serves an important role in inducing apoptosis in malignant cells^22–26^.

The effectiveness of trailed non-thermal treatment is cell dependent, meaning alterations to the treatment process may yield better results for different types of cancer. This characteristic could make future treatment more target specific and customisable to a patient’s needs. Non-thermal treatment has been found to be dilatability dependent allowing dose size to be varied adding to the custom ability of the treatment^27^. Large amounts of anti-cancer research have been conducted with two different designs of DBD non-thermal plasma reactors, these are named the Atmospheric Pressure Plasma Jet (APPJ) and the DBD reactor^26^. The DBD reactor, which is the subject of this study, has several advantages over the jet reactor. DBD’s do not require a carrier gas as they produce plasma in air and directly across the treated sample itself. However, one major drawback to the DBD reactor is that it requires an electrode to be placed behind the treated sample, which in a surgical environment this could prove to be more intrusive and has the potential of more complications in the treatment processes.

Homogeneous and filament plasma regimes arise from the behaviour and the arcing characteristics of the plasma discharge. Homogeneous discharges are sought after in medical trials as they provide a constant and even plasma dose for experimentation^28^. Reactor design is vital to this characteristic as less favourable filamentary plasma can be produced. The characteristics of the plasma regimes depends on several variables including voltage source (DC/AC/nano/micro-pulse), excitation frequency, voltage amplitude, reactor design and carrier gas. DBD plasma, which is excited by short pulsed high voltage, in the order of nanoseconds, can enhance plasma chemistry while maintaining low gas temperature. Plasma chemistry is enhanced as this plasma can produce increased RONS. Research found that using ns-DBD plasmas increased gas temperature by 20% but OH production by 100–500%^29^.

Investigating new methods for the treatment of leukaemia and other blood cancers is very important as blood serves many purposes within the body such as, inflammatory response and blood coagulation. The first study to examine the effects of non-thermal plasma on leukaemia cells was conducted by Barekzi and Laroussi^30^. A DBD plasma pencil was used to treat human T-cell line from a paediatric patient diagnosed with acute lymphoblastic leukaemia. Findings showed an accelerated rate of cell death between 3 and 4 min of plasma exposure. Interestingly, they observed that when the samples were analysed at 12, 36 and 60 h after treatment, the cell death rates were statistically increasing with longer treatment exposure times. Additionally, a single dose of plasma treatment continued to have apoptotic effects on cancer cells even 60 h after treatment. Collectively, suggesting that cell death rates are closely governed by the treatment exposure duration, thus being indicative of a time-dependent response. Although non-thermal plasma was found to effectively induce apoptosis in several cancer lines such as leukaemia however, their effectiveness is limited and can be improved. The aim of the current investigation is to characterise the effect of different parameters on DBD reactors effectiveness on plasma induced leukaemia cell death.

## Methods

### Plasma generation, and voltage and current measurements

**Figure 1:**
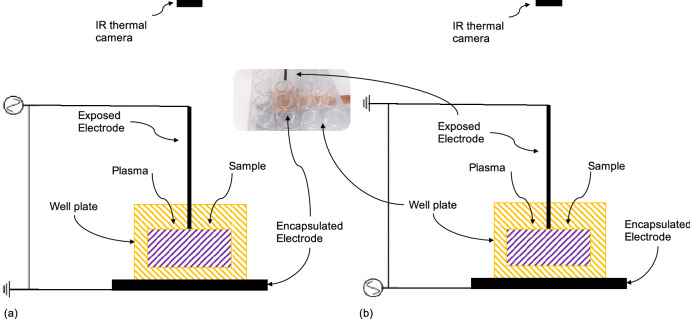
Schematic of reactor designs and experimental setup. (**a**) ns-DBD$^+$, (**b**) ns-DBD$^-$.

The ns-DBD reactor (as called previously by Shashurin et al.^31^) is shown in Fig. 1. The ns-DBD reactor uses a well plate as a dielectric barrier and it has a solid 2 mm rod as an exposed electrode and copper tape as encapsulated electrode. Polarity effect was examined by reconnecting the wires to electrodes. ns-DBD reactors are named ns-DBD$^+$, when exposed rod electrode was connected to the power supply, Fig. 1a, and ns-DBD$^-$, Fig. 1b, when exposed rod electrode was earthed. The height of the well plate is 20 mm and the distance from the surface of the cell medium to the edge of the well plate is about 8 mm. The distance from the electrode to the liquid is tried to be minimum and it is estimated to be around 1 mm. The thickness of the bottom wall of the well plate which acts as dielectric material is 3 mm. The gap between electrodes is filled by polystyrene (wall) and cell solution. Cells were directly exposed with plasma while untreated cells were used as negative control.

In order to enhance the plasma chemistry for this investigation, a nanosecond pulse high-voltage generator (Megaimpulse, model: NPG-18/3500(N)) with a pulse rise time of approximately 4 ns was used. This is different to ac-driven DBD (ac-DBD) plasma, which is excited by sinusoidal signal. The output voltage and frequency from the transformer was measured using a LeCroy PPE-20kV high voltage probe that was provided with a detailed calibration from the manufacturers. The output current was measured using a current shunt cs-10/500. The voltage and current output signals were connected to a LeCroy 500 MHz wavesurfer 3054, 4 GS oscilloscope where the signals were displayed and recorded.

### Temperature measurement and control

The thermal properties of the reactor as a result of plasma generation can be explored using Infra-Red thermal imaging, see Fig. 1 for thermal camera location. A FLIRONE PRO LT IR camera was used with a thermal overlaid picture. The reactor was switched on for 2 s to reach the stable run set before commencing the treatment. Constant IR camera monitoring ensured that the temperature of the sample was kept to 35 ± 1 degree during the experiments. Experiment was halted when temperature exceeded the interval, thus letting the sample cool down to 34 degrees before resuming it.

### Cell culture and measurement of cell viability

Jurkat cells and U937 cells were cultured in RPMI 1640 growth medium (Lonza, UK) along with 10% foetal bovine serum, 100 IU/ml penicillin and 100 $\mu$g/ml streptomycin and 2 mM L-glutamine solution (All purchased from Sigma-Aldrich Company Ltd., Dorset, UK). Cultured cells were maintained in a humidified atmosphere containing 5% CO$_2$ at 37 °C. Subculture of the cells occurred every three days in order to expand the concentration of cells. 1 $\times$ 10$^6$ cells/ml in 2 ml complete medium were directly exposed to plasma treatment in a 12-well cell culture plate. Jurkat and U937 cells maintained under the same culture conditions without exposure to plasma were used as negative controls.

Cell viability was measured at 2 and 24 h after plasma exposure using trypan blue exclusion assay. Some experiments were performed with 6 mM N-acetyl-L-cysteine (NAC) (Sigma-Aldrich) pre-treatment for 1 h before cells were exposed to plasma. Plasma treated or untreated cells were then mixed with equal volume of 0.4% trypan blue solution (Sigma-Aldrich Company Ltd) before loading into C-Chip disposable counting chamber (NanoEnTek, USA). Both viable (unstained) and non-viable (stained) cells were counted under microscope. Total number of cells were number of viable cells plus non-viable cells. The percentage of viable cells was calculated using Equation (1).

1 $$\begin{aligned} \% \ viable \ cells = \frac{number \ of \ viable \ cells}{total \ number \ of \ cells} \times 100 \end{aligned}$$

Various conditions of plasma exposure were tested to achieve IC50 response (the effectiveness of 50% cell viability).

### MTT assay

Jurkat and U937 cells were seeded into a 96-well plate immediately after treatment with DBD$^+$ or DBD$^-$ plasma. After 24 h post-treatment culture, the CellTiter 96 Non-Radioactive Cell Proliferation Assay solutions (Promega, UK) were used to analyse viable cells. Briefly, 15 $\mu$ l of dye solution was added to each sample in a 96-well plate. The plate was incubated for 4 h at 37 °C and 5% CO$_2$. Stopping solution 100 $\mu$ l was then added to each sample and the absorbance of 570 nm was measured using a spectrophotometer.

### Cell apoptosis assay

Cell apoptosis and necrosis were evaluated at 4 h after plasma exposure using the Apoptosis/Necrosis Detection Kit (Abcam, UK) according to the manufacturer’s instructions. Phosphatidylserine binding to Apopxin Deep Red (APC) (Ex/Em = 630/660 nm) indicated apoptotic cells. DNA Nuclear Green, DCSI (Ex/Em = 490/525 nm), a membrane-impermeable dye, staining the nucleus of damaged cells, indicated late apoptotic and necrotic cells. Plasma treated or untreated cells were collected and measured on a MACSQuant Analyser 16 (Miltenyi Biotec, Germany). The data were analysed using MACSQuantify software 2.13 (Miltenyi Biotec, Germany).

### Detection of intracellular ROS

The reactive oxygen species (ROS) in Jurkat and U937 cells were assessed at 1 h after plasma exposure. Plasma treated or untreated cells were collected and stained with CellROX Green Reagent (ThermoFisher Scientific, UK) at a concentration of 5 $\mu$M and incubated for 30 minutes at 37 °C. Cells were washed three times with PBS. The fluorescence resulting from CellROX oxidative stress reagents were measured at 485/520 nm (excitation/emission) using MACSQuant Analyser 16.

### Real-time PCR

Total RNA of Jurkat cells were isolated with the EZ-RNA II total RNA isolation kit (BI, Biological Industries, Israel) according to manufacturer’s instructions at 24 h after plasma exposure. Quantification of RNA was carried out using a Nanodrop 2000 (Thermofisher Scientific). cDNA was synthesised by reverse transcriptase reaction using SensiFAST cDNA Synthesis Kit (Bioline, UK). Polymerase chain reactions were carried out using SYBR Green Master Mix (ThermoFisher Scientific) and specific primers (Sigma-Aldrich) for *P53* (NM_001126114.1), *BAD* (NM_004322.3), *BID* (NM_197966.2) and *GAPDH* (NM_002046.3). The expressions of GAPDH were used as endogenous controls for normalisation. The changes in mRNA expression of *P53*, *BAD* and *BID* were analysed using the threshold cycle 2$^{-\Delta \Delta Ct}$ method.

### Caspase 9 activity assay

Jurkat cells were seeded into a 96-well white walled plate immediately after plasma treatment. After 4-h incubation, the caspase 9 activities in plasma treated and untreated cells were measured using the Caspase-Glo 9 Assay kit (Promega) according to the manufacturer’s instructions. Luminescence from each sample was measured using Gen5 Microplate Reader (BioTek, UK).

### Extracellular H$$_2$$O$$_2$$, NO$$_2$$$$^-$$ and NO$$_3$$$$^-$$ assays

The extracellular H$_2$O$_2$ concentrations in Jurkat and U937 cell culture media were assessed at 1 h and 24 h after plasma exposure. The Hydrogen Peroxide Assay Kit (Abcam) was used according to manufacturer’s instruction. Cell culture media samples were collected, centrifuged and deproteinization following the protocol. The H$_2$O$_2$ concentration in the samples were calculated based on the standard curve. The concentrations of nitrite and nitrate (NO$_2$ $^-$ and NO$_3$ $^-$ ) in Jurkat cell culture media were measured at 1 h and 24 h after plasma exposure using the Nitric Oxide Assay Kit (ThermoFisher Scientific, Austria) according to manufacturer’s instruction.

### Statistical analysis

An arithmetic mean value represented an average for each experiment ± standard deviation calculated from three independent experiments. Two-tailed t-test was used to compare the control (plasma untreated cells) with the test samples. Significance is captured at *p*-value $\le$ 0.05.

## Results and discussion

### DBD reactor treatment

**Figure 2:**
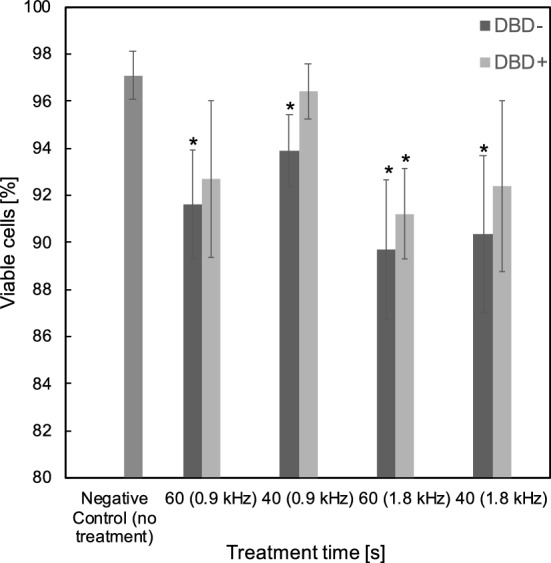
Effects of time, frequency, and polarity on cell viability using the ns-DBD reactor. Results are presented as the mean ± SD (n = 3). Student’s t-test was preformed, and the significance is indicated as *$p<0.05$.

An investigation into the effects of polarity, treatment time and frequency on the effectiveness of plasma treatment was conducted using the ns-DBD reactor. The treatment times used were 40 and 60 s at a frequency of 0.9 and 1.8 kHz. The voltage was constant at 12 kV. Results from Fig. 2 show similar trend of reduction on cell viability by increase of treatment time and increase of driven frequency. A noticeable trend can be seen in the effects of reactor polarity on cell viability. The ns-DBD$^-$ treatment has an increased effect at the higher frequency oppose to the ns-DBD$^+$ scenario, both tend to increase cell death at higher exposure time. *P*-value < 0.05 uncertainty was achieved for the majority of the results compared to the negative control (0 exposure) which are indicated by an asterisk in the figure. This may be due to the radical charged species production and their movement through the sample by electrostatic forces. When one of the electrodes in the configuration is provided with sufficient voltage and frequency magnitude, the breakdown field strength of the gas in the discharge gap has been reached, resulting in the formation of a plasma that appears constant to the naked eye^1^. The negative electrode of a reactor provides a source of electrons contrary the positive provides a well that can mitigate electrons. The direction of the transfer of electrons from one electrode to the other across the sample and dielectric barrier produces a different ion producing mechanism therefore altering the effectiveness of treatment. From the investigation, it shows that electron production on the sample side of the dielectric barrier provides a more effective treatment. The permeability of the dialectic material used in a reactor also influences the electron transfer mechanism through the sample.

#### Cell viability at 2-h and 24-h post treatment

**Figure 3:**
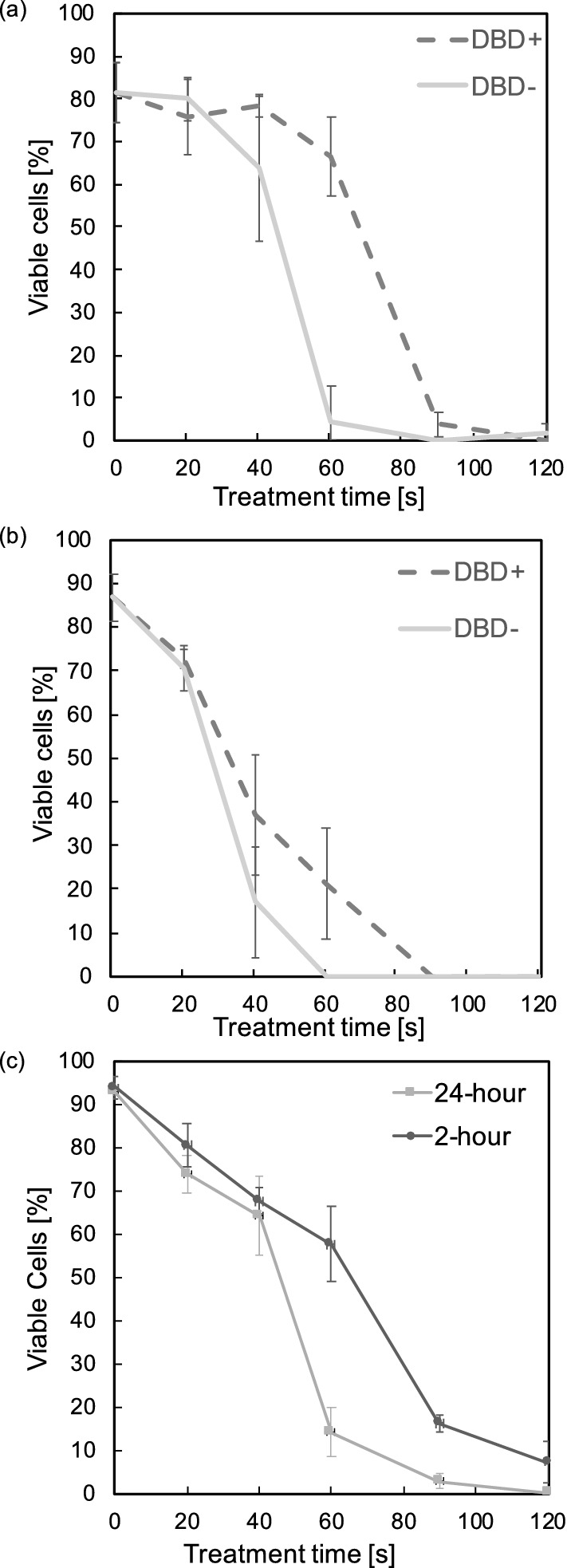
Cell viability versus exposure time (**a**) 2-h after treatment for Jurkat Cell, (**b**) 24-h after treatment for Jurkat cell, and (**c**) 2-h and 24-h after treatment for U937 Cells by ns-DBD$^-$. All results achieved a $p<$0.05 compared to the negative control (0 exposure), (n = 3).

Following previous investigations, the effects of plasma treatment were significant but unable to reach a minimum threshold in order to define an IC50 value. Longer exposure to plasma and increased intensity was required to reach a minimum cell viability plateau. Exposure to treatment was increased (20, 40, 60, 90 and 120 s) and cell viability measured 2 h after treatment for both the ns-DBD$^+$ and ns-DBD$^-$ reactors. Voltage and frequency were fixed at 17 kV and 3.5 kHz, respectively. Figure 3a displays result following this investigation. The graph shows significant effects on cell viability after 40 s of exposure with a minimum threshold of 0% viability reached. From the results the that the IC50 for the ns-DBD$^-$ will occur between 40–60 and 60–80 s for the ns-DBD$^+$ reactor.

The same cell viability assay was conducted 24 h after the treatment. Figure 3b demonstrates that cell death was still occurring 24 h after treatment. This could be due to radicals remaining in the media or delayed apoptosis in some cells. The negative control (0 exposure) cell viability during this investigation increased from Fig. 3a by around 4%. This is due to the availability of nutrients within the media allowing continued cell growth. Similar biological effects on the viabilities of U937 cells were observed at 2 h and 24 h after plasma treatment with ns-DBD$^-$ reactor (Fig. 3c).

#### 24-h post treatment MTT assay

**Figure 4:**
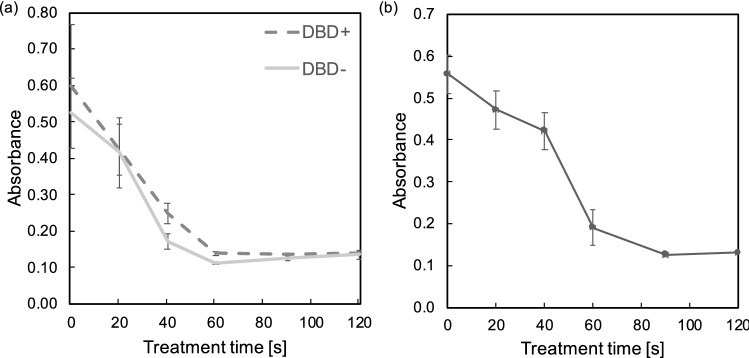
Absorbance of 570 nm spectrum 24 h after treatment. (**a**) Jurkat cell, (**b**) U937 Cell treated by ns-DBD$^-$. Results from 40 s onward achieved a $p<0.05$ (n = 3).

The results of MTT assays conducted on the 24-h post treatment of Jurkat and U939 cells (Fig. 4) confirmed the observations in the trypan blue assays (Fig. 3b,c). 570 nm absorbance was measured using a spectrophotometer. CellTiter 96 was used during this assay as a reagent. Absorbance is indicative of enzyme activity and general cell viability. It demonstrates that the leukemia cell viability decreased significantly in vitro when the duration of the plasma treatment increased.

#### Types of cell death following plasm treatment

**Figure 5:**
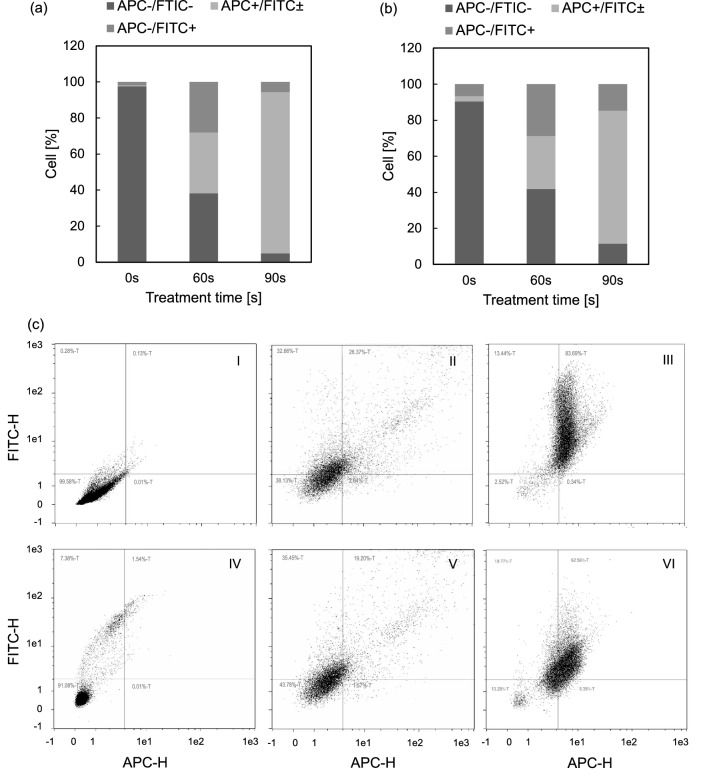
Analysis of apoptosis and necrosis induced by plasma in leukaemia cells in vitro. (**a**) Jurkat cells and (**b**) U937 cells were assessed at 4-h after treatment with ns-DND$^-$ plasma. Data represent mean percentage (%) of live (APC-/FITC-), apoptotic (APC+/FITC±) and necrotic cells (APC-/FITC+), n = 3. Compared to untreated cells (0 exposure), significant percentages of apoptotic and/or necrotic cells exist in plasma treated cells vs control cells ($p<0.05$, n = 3). (**c**) Representative dot plots of apoptosis and necrosis results using APC (Apopxin labelling phosphatidylserine, red fluorescence) vs FITC (Nuclear green DCS1 labelling the nucleus of damaged cells, green fluorescence). I–III, Jurkat cells treated with plasma for 0, 60, 90 s respectively; IV–VI, U937 cells treated with plasma for 0, 60, and 90 s, respectively.

To identify the types of leukemia cell death induced by plasma treatment, flow cytometry was performed. Figure 5 shows that both apoptosis and necrosis were induced in Jurkat and U937 cell by plasma treatment. The percentages of vital cells (APC-/FITC-) were significantly reduced in plasma treated cells compared to untreated cells. These results are consistent with previous findings from cell viability assays by us (Fig. 3) and other research groups^19^. It was found that 90 s plasma treatment induced higher proportion of apoptotic cells (APC+/FITC±) than necrotic cells (APC-/FITC+). However, there was no significant differences in the percentages of apoptotic and necrotic cells after 60 s plasma treatment for 4 h. It indicates plasma could induce both apoptosis and necrosis of leukaemia cells in vitro.

#### Effects of plasma on the expressions and activity of apoptosis/necrosis regulatory molecules and on the intracellular ROS and extracellular H$$_2$$O$$_2$$, NO$$_2$$$$^-$$ and NO$$_3$$$$^-$$ levels

**Figure 6:**
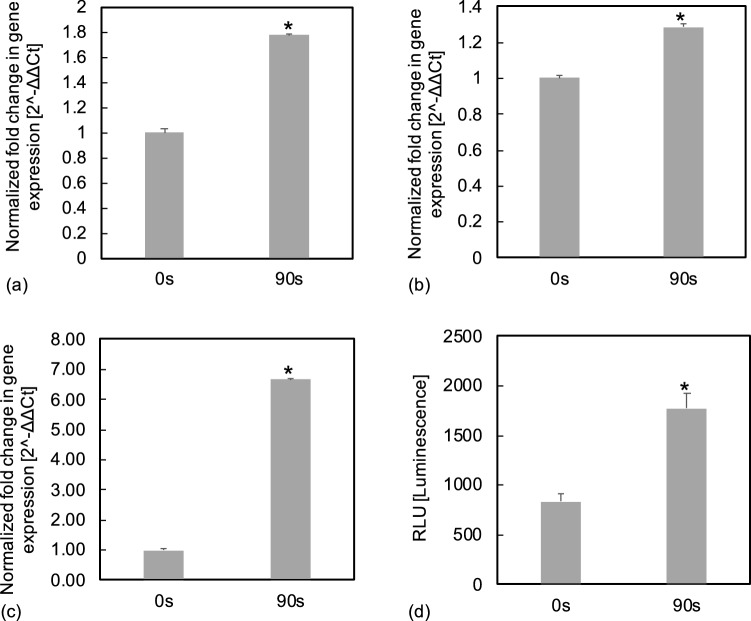
(**a**)–(**c**) Real-time PCR analysis of alterations of mRNA expressions in Jurkat cells after plasma treatment in vitro. Increased expressions of (**a**) *P53*, (**b**) *BID*, and (**c**) *BAD* following plasma treatment was shown as mean of fold-changes *vs* untreated controls (0 s exposure). The expression of housekeeping gene *GAPDH* was used for normalisation. (**d**) The caspase 9 activities in plasma treated and untreated Jurkat cells were measured. The luminescent signal (RLU) generated was proportional to the amount of caspase 9 activities present. Data represent mean of independent tests (n = 3). (**a**)–(**d**) show significant differences in mRNA expression and caspase 9 activity between plasma treated cells vs the control ($p<0.05$).

To explore the mechanisms of plasma exposure on the induction of leukemia cell death in vitro, real-time PCR analyses were used to evaluate mRNA expressions of apoptosis/necrosis regulatory molecules. *P53* expression was significantly up-regulated 1.78-fold in plasma-treated Jurkat cells when compared with untreated controls. Upregulations of *BID* (1.28-fold) and *BAD* (6.67-fold) were also identified in plasma-treated Jurkat cells (Fig. 6a–c). Caspase 9 plays a key initiator role in the intrinsic apoptotic pathway^32^. The Bcl-2 family members BAX and BAK undergo oligomerization in the outer mitochondrial membrane resulting in the release of apoptosis inducing substrates and the activation of caspases^32^. In this study, the enzymatic activity of caspase 9 was significantly enhanced in plasma-treated Jurkat cells in comparison with untreated control cells (Fig. 6d). It was reported that caspase 9 can cleave BID and this cleavage of BID is required for ROS production^33^.

**Figure 7:**
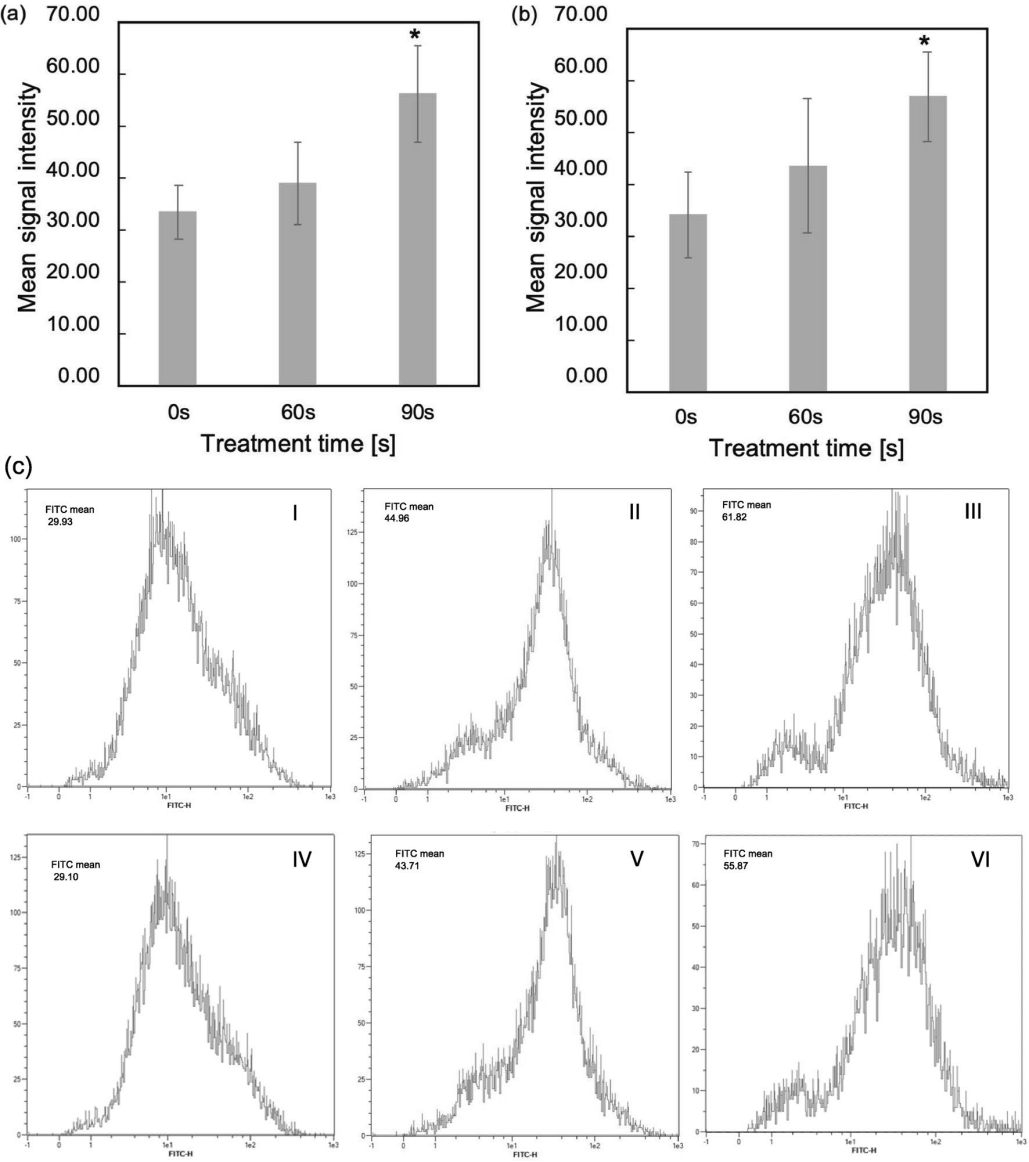
Detection of intracellular ROS levels in Jurkat (**a**) and U937 cells (**b**) after plasma treatment. Quantitation analysis of oxidative stress based on staining with CellROX Green Reagent (Ex/Em = 485/520 nm) and flow cytometry. The mean signal intensity was proportional to the amount of intracellular ROS. Data represent mean of independent tests (n = 3). (**c**) Representative histograms of FITC fluorescence intensity in log scale show intracellular ROS levels in Jurkat (I–III) and U937 cells (IV–VI) following ns-DBD$^-$ plasma 0, 60, 90 s treatment respectively.

The effects of plasma on the levels of intracellular ROS in Jurkat and U937 cells were evaluated at 1 h after plasma treatment by flow cytometry, (Fig. 7a,b). Representative histograms of FITC fluorescence intensities indicating intracellular ROS levels in plasma-treated cells are shown in Fig. 7c. Figure 7 demonstrates higher intracellular ROS levels after plasma treatment in both cell lines; however, only 90 s plasma exposure significantly increased intracellular ROS levels. These results support the observations in the cell viability and apoptosis tests (Fig. 3 and Fig. 5, respectively).

**Figure 8:**
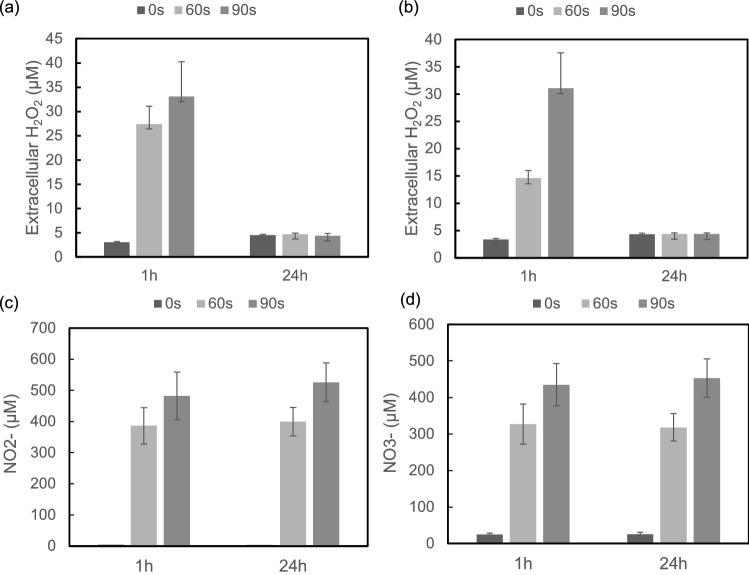
Detection of extracellular H$_2$O$_2$, NO$_2$ $^-$ and NO$_3$ $^-$ levels in cell culture media after plasma treatment at 1 h and 24 h. H$_2$O$_2$ concentrations in (**a**) Jurkat and (**b**) U937 cell culture media were measured following plasma treatment for 0, 60 and 90 s respectively. The concentrations of NO$_2^-$ (**c**) and NO$_3^-$ (**d**) in Jurkat cell culture media were measured at 1 h and 24 h following plasma treatment. All data represent mean concentration ± SD, n = 3. Student’s t-test was preformed comparing plasma treated samples vs untreated control and the significance is indicated as * $p< 0.05$.

Plasma can generate a different kind of free radicals that react with the cell culture medium to form long-lived stable reactive species such as H$_2$O$_2$, NO$_2$ $^-$ and NO$_3$ $^-$. The extracellular levels of H$_2$O$_2$ in both Jurkat and U937 cell media increased significantly at 1 h of plasma treatment compared to the untreated control (Fig. 8a,b). Plasma treatment for 90 s induced more extracellular H$_2$O$_2$ generation than the 60 s treatment. However, after 24 h extracellular H$_2$O$_2$ in plasma-treated cell media descended to the similar levels as in plasma-untreated controls. The levels of NO$_2$ $^-$ and NO$_3$ $^-$ in Jurkat cell media increased significantly after plasma treatment at 1 h and 24 h (Fig. 8c,d). There were no significant changes of extracellular NO$_2$ $^-$ and NO$_3$ $^-$ concentrations between 1 and 24 h. The fast consumption of plasm-produced H$_2$O$_2$ in cell medium by cancer cells have been observed previously^27,34^. The rise of intracellular ROS observed in Fig. 7 may be partially attributed to the diffusion of extracellular ROS and intracellular formation. The changes of transmembrane proteins, aquaporins, following plasm treatment have been reported^35^. Specific aquaporins involved in the transportation of reactive species across leukemia cell membrane and the underlying mechanisms of the transport of need further study. Previous study demonstrated that the extracellular NO$_2$ $^-$/NO$_3$ $^-$ at concentrations of 900 $\mu$M had no effects on cancer cell viability^34^. Although plasma treatment induced NO$_2$ $^-$ and NO$_3$ $^-$ generation in Jurkat cell media, however, whether these species efficiently permeated cellular membrane as H$_2$O$_2$ did and whether they play significant roles in the plasma-mediated leukaemia cell viability remain elusive.

**Figure 9:**
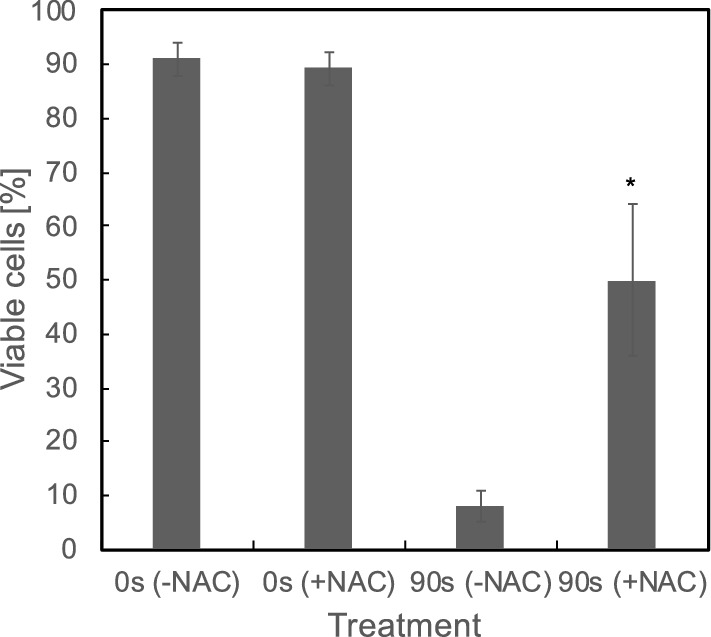
NAC counteracts the cytotoxic effect of plasma on Jurkat cells. Jurkat cells pre-treated with/without NAC for 1 h, then treated with ns-DBD$^-$ plasma for 90 s. cell viability were tested 2 h following plasma exposure. Results are presented as the mean ± SD (n = 3). The significance is indicated as *$p<$ 0.05.

Previous studies have demonstrated that pre-treating cancer cells with the intracellular ROS scavengers could counteract the anti-cancer effects of plasma treatment^19,27,36,36^. To investigate whether the rise of intracellular ROS plays a crucial role in cytotoxic effect of plasma on leukaemia cells, Jurkat cells were pre-treated with 6 mM NAC for 1 h and then with ns-DBD$^-$ plasma for 90 s. After 2 h from plasma treatment, a significant increase in cell viability was observed in cancer cells pretreated with NAC and then exposed to plasma compared to cells treated only with plasma, see Fig. 9. This is in accordance with previous reports that ROS is one main mediator that induce cell death by plasma^26,37^. However, the cytotoxic effect of plasma in Jurkat cells was not completely antagonized by NAC. Previous study by other researchers found that NAC did not fully abolish phosphorylation of H2A.X, a marker of genotoxicity^19^, and plasma could induce ROS-independent cell death^38^. All these indicate that other factors except intracellular ROS involved in plasma-induced cell death.

The cell death mechanisms induced by plasma treatment have been investigated in different types of cancer cell lines include leukemia. The chemical factors are largely regarded to cause cell apoptosis by both direct and indirect plasma treatment^21,26,30^. In this study, necrosis was observed in both plasma-treated cell lines. Recently, it was demonstrated that the physical factor, electromagnetic waves in plasma, might cause a new type of necrosis in glioblastoma cells^39^. Our results could suggest that both physical and chemical factors of plasma contributed to leukemia cell death in vitro. The detailed mechanisms triggering leukaemia cell death and especially ROS-independent cytotoxicity need further investigation.

Although the underlying mechanisms of plasma-induced leukemia cell death remain unclear, results from this study and other previous studies^12,16,17,19^ strongly suggest that excessive production of oxidative stress, exceeding the cellular anti-oxidative defense, can activate both apoptosis and necrosis singling pathways and lead to leukemia cell death. The apoptosis regulatory molecules such as p53, BAD, BID and caspase 9 may play vital roles in plasma caused cell death.

The use of plasma for the treatment of hematological malignancies is a novel field in clinical oncology. The marked anti-leukaemia effects induced by plasma treatment in this study and others^17,19,30^ highlight its therapeutic potential. Given plasma could enhance drug delivery and enhance the effects of standard chemotherapy and even in resistant cancer cells^40,41^, plasma may be applied as part of a combination therapy in leukaemia treatment.

### Plasma regime and thermal properties of plasma discharges

**Figure 10:**
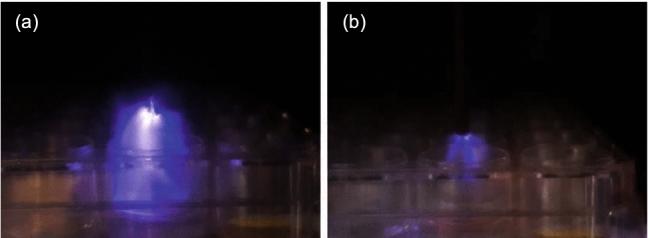
Plasma discharge generated in (**a**) ns-DBD$^+$ reactor, and (**b**) ns-DBD$^-$ reactor.

The images presented in Fig. 10 show the plasma discharge regimes for the positive (a) and negative (b) ns-DBD reactors. The generated plasma regimes are vastly different. The ns-DBD$^+$ reactor produced highly filamented plasma that domed towards the negative electrode. The plasma produced emits more light than that of the ns-DBD$^-$ reactor. The regime of the negative reactor is homogenous but with the lower light emittance less can be seen.

**Figure 11:**
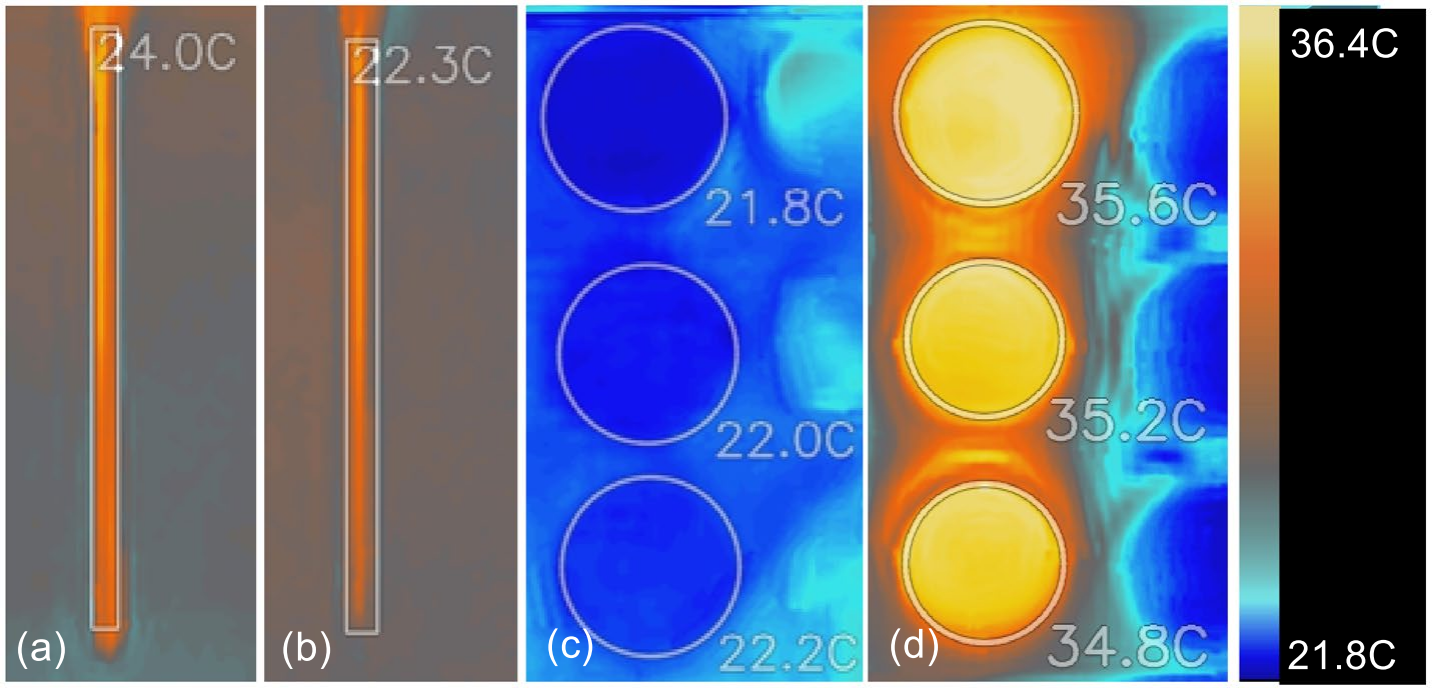
Representative temperature from IR thermal imaging of: (**a**) ns-DBD$^+$ exposed electrode, (**b**) ns-DBD$^-$ exposed electrode, (**c**) samples before plasma treatment, and (**d**) samples during plasma treatment.

The temperature measured at thermal hotspots is displayed on each image of Fig. 11. The average temperature of exposed electrodes of ns-DBD$^+$ and ns-DBD$^-$ during experiment are recorded in Fig. 11a,b, respectively. The ns-DBD$^+$ reactor produced a slightly higher temperature of 24 degrees compared to the 22.3 degrees of ns-DBD$^-$. However, this is not representative of the temperature of the treatment sample (medium with cells). Figure 11c shows the temperature of the samples before plasma treatment. It has been noticed that the temperature of the samples increased to temperatures as high as 50 degrees for both reactors during the experiment. This is different from what has been reported in literature using ac-DBD where only slight increase in the temperature of medium observed^37,42^. To avoid the influence of temperature on our treatment, the temperature of the samples has been kept at 35 $\pm 1$ degree by constant monitoring of the temperature during the experiment, see Fig. 11d.

### ns-DBD electrical characteristics

**Figure 12:**
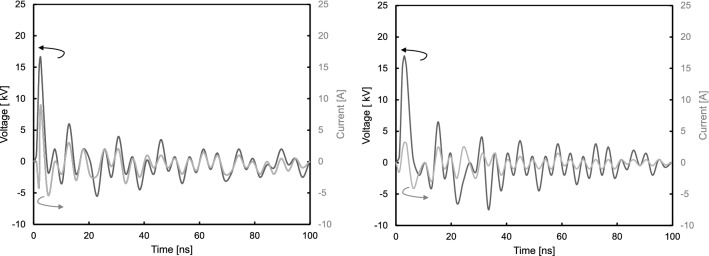
Voltage and current characteristics of the ns-DBD$^-$ (**a**) and ns-DBD$^+$ (**b**) reactors.

The electrical characteristics of the ns-DBD reactors were measured using the experimental setup section. Figure 12 shows the first pulses of voltage and current. When one of the electrodes in the configuration is provided with sufficient voltage magnitude, current peak will appear in the current trace. It is different from ac-DBD that microdischarges could be seen in the current trace and usually, the amplitude is within a few mA (or few tens mA) levels. The current discharges occur due to reaching the break down field strength of the gas in the discharge gap, resulting in the formation of a plasma that appears constant to the naked eye. The microsecond pulse (ac-DBD) has relatively low dU/dt. Therefore, the voltage rises slowly on the HV electrode, the discharge occurs occasionally, maybe after some delay, and non-uniform over the electrode area. The barrier improves the uniformity and prevents arcing; therefore, it is required obligatory. The discharge current is limited by the barrier. In contrast, nanosecond pulse has much higher dU/dt. So, much higher over-voltage can be obtained on the electrodes before the breakdown. This over-voltage helps to get the breakdown. The current through the reactor is also much higher due to high dU/dt. In other words, many mA level spikes in the case of microsecond DBD are replaced by a single (of few) much more powerful spike(s) over the electrode area.

The ns-DBD$^-$ reactor is seen to have a higher maximum current than the ns-DBD$^+$ setup. The maximum positive current was found to be 9 A and 4 A respectively for the reactors. This current has a duration of 2e$^{-7}$ s as this is half the period of the nanosecond pulse. Negative spike on both current oscillograms before the main pulse is a result of bad grounding the cable from the current shunt to oscilloscope. However, as it presents on both graphs, it makes the comparison possible.

Based on the current and voltage measurements, the power consumption by the actuator is calculated using Equation (2), where T and N represent the time period and the number of cycles, respectively.

2 $$\begin{aligned} Power = \frac{1}{N T}\int _{NT} V(t).I(t) dt \end{aligned}$$

The ns-DBD$^-$ reactor has a higher maximum current of 9 A, this occurred on the first driven nanosecond pulse. In this investigation the power consumption is calculated to be almost 65% more for the ns-DBD$^-$ compare with that of ns-DBD$^+$ reactor. As the driven voltage forms should be matching the maximum current can be seen to be much larger in the ns-DBD$^-$ therefore having a higher power. Not having access to all the data, this calculation does only consider an estimation and therefore is not representative of the true power consumption.

## Conclusion and future work

Investigations into reactor designs on the effectiveness of nanosecond-pulsed plasma treatment have shown a large variance in performance. ns-DBD reactors displayed greater promise especially for longer exposure periods. The effect of polarity on plasma discharge regime was significant and this also influenced the effectiveness of treatment. The delayed cell response to plasma treatment from ns-DBD$^+$ and ns-DBD$^-$ reactors shown by the 2-hour and 24-hour post treatment results. Polarity changes proved to have a substantial effect on treatment, this must be evidence of a difference plasma regimes production, homogeneous versus filamentary, that as a result yield in different radical ion production due to the direction of electron discharges.

Our results demonstrate that the plasma produced by ns-DBD- could induce both apoptosis and necrosis of human Jurkat and U937 cells, and this cytotoxic effect of plasma was not completely antagonized by N-acetyl cysteine. It indicates that plasma could induce ROS-independent cell death. Gene expression analyses revealed that p53, BAD, BID and caspase 9 may play vital roles in plasma caused cell death.

Results confirms the custom ability of plasma hematologic cancer therapy. Multi-objective optimisation techniques can be employed to promote the development of such reactors^2,43,44^. If in future a similar type of reactor is used for blood cancer treatment, some sort of bypass machine would be required as well as the need for a standardised plasma source which can make it cumbersome for treatment. To overcome this drawback, plasma-activated liquids (PAL) or plasma-activated cell media (PAM) are studied in literature.Characterising the power and discharge of a ns-DBD shows that ns-DBD$^-$ consume more power than ns-DBD$^+$ which can be due to the filamentary regimes presents in later reactor.

Further investigation into other reactor designs could harbour more potential and improve effectiveness of nano-plasma treatment while reducing invasiveness. Testing on different types of leukemia cell lines, primary leukemia cells as well as healthy cells to understand the effective treatment thresholds would be of great significance for future research. On top of this, research into radical species production mechanisms and the possible addition of electromagnetic devices to plasma reactors could improve the delivery of radical species to targeted areas.

## Supplementary Information

Supplementary Information.
