# Supplementary material for: Nanosecond-pulsed DBD plasma treatment on human leukaemia Jurkat cells and monoblastic U937 cells in vitro
Source: Sci Rep. 2022 Apr 15;12:6270. doi: 10.1038/s41598-022-10056-8 (PMC9012873; doi:10.1038/s41598-022-10056-8)
Supplement: Supplementary file 1 — Supplementary Information. [file 41598_2022_10056_MOESM1_ESM.pdf]

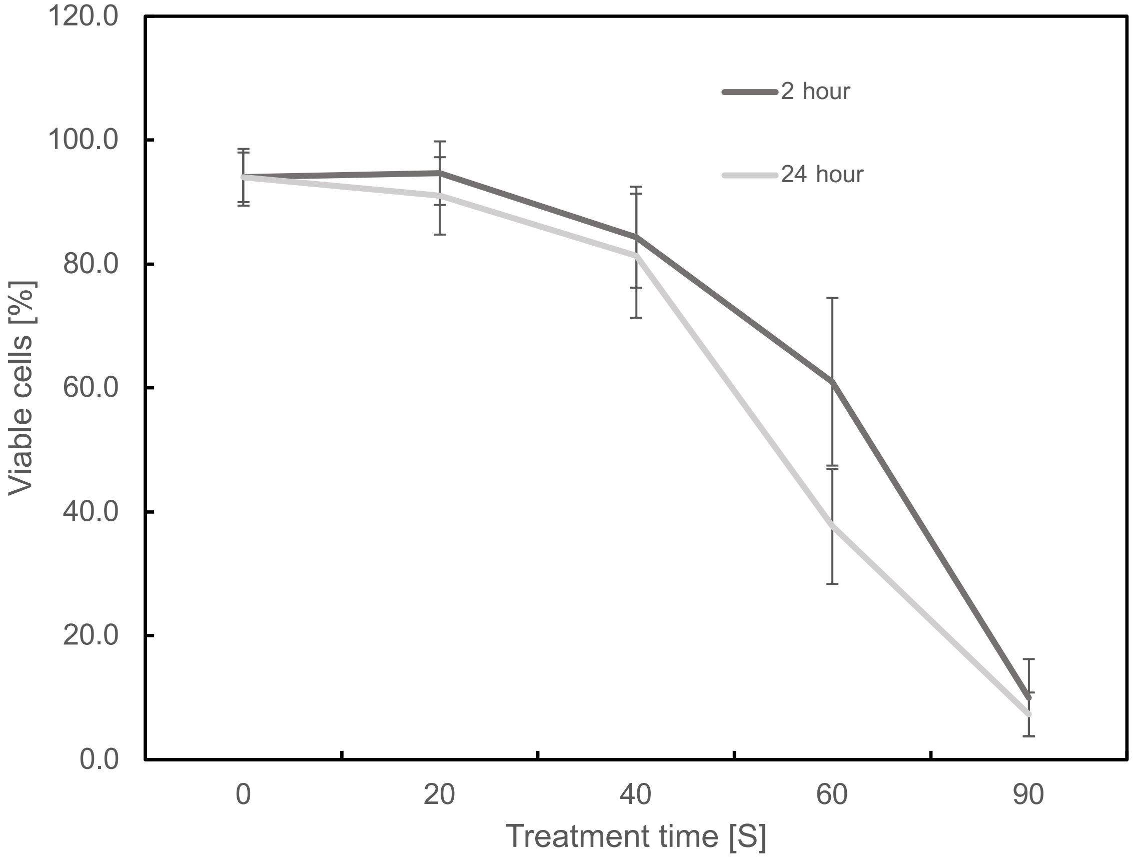

Supplementary Figure: THP1 cell viability versus ns-DBD- plasma exposure time. Cell viability were tested 2 and 24 hours following plasma treatment. Results are presented as the mean ± SD (n=3). The 60s and 90s exposure to plasma significantly ($p < 0.05$) reduced cell viability compared to the negative control (0 s exposure).
